# Nonadditive and allele-specific expression of ghrelin in hybrid tilapia

**DOI:** 10.3389/fendo.2023.1292730

**Published:** 2023-12-13

**Authors:** Huan Zhong, Bingxin Ren, Chenyi Lou, Yi Zhou, Yongju Luo, Jun Xiao

**Affiliations:** ^1^ State Key Laboratory of Developmental Biology of Freshwater Fish, College of Life Sciences, Hunan Normal University, Changsha, China; ^2^ Tilapia Genetics and Breeding Center, Guangxi Academy of Fishery Sciences, Nanning, China

**Keywords:** ghrelin, hybrid tilapia, nonadditive expression, gene expression, heterosis

## Abstract

**Background:**

Interspecies hybridization is an important breeding method to generate fishes with heterosis in aquaculture. Using this method, hybrid Nile tilapia (*Oreochromis niloticus*, ♀) × blue tilapia (*Oreochromis aureus*, ♂) has been produced and widely farmed due to its growth and appetite superiorities. However, the genetic mechanism of these advanced traits is still not well understood. *Ghrelin* is a crucial gene that regulates growth and appetite in fishes. In the present study, we focused on the expression characteristics and its regulation of *ghrelin* in the hybrid.

**Results:**

The tissue distribution analysis showed that *ghrelin* was predominantly expressed in the stomach in the hybrid. *Ghrelin* was more highly expressed in the stomach in the hybrid and Nile tilapia, compared to blue tilapia, showing a nonadditive pattern. Two single-nucleotide polymorphism (SNP) sites were identified including T/C and C/G from the second exon in the *ghrelin* gene from Nile tilapia and blue tilapia. By pyrosequencing based on the SNP sites, the allele-specific expression (ASE) of *ghrelin* in the hybrid was assayed. The result indicated that *ghrelin* in the hybrid showed higher maternal allelic transcript ratios. Fasting significantly increased *ghrelin* overall expression at 4, 8, 12, 24, and 48 h. In addition, higher maternal allelic transcript ratios were not changed in the fasting hybrids at 48 h. The *cis* and *trans* effects were determined by evaluating the overall expression and ASE values in the hybrid. The expression of *ghrelin* was mediated by compensating *cis* and *trans* effects in hybrid.

**Conclusion:**

In summary, the present lines of evidence showed the nonadditive expression of *ghrelin* in the hybrid tilapia and its regulation by subgenomes, offering new insight into gene expression characteristics in hybrids.

## 1 Introduction

Fish farming provides abundant proteins for people around the world. In recent years, genetic breeding has been accelerating the yields and quality of the farmed fish. Among the breeding technologies, selective breeding and hybridization have been widely used to improve the fish germplasm (1). Compared to selective breeding, hybridization combined the genetic resources from different lineages from the same species (intraspecific hybridization) or even different species (distant hybridization) (2). Hybridization may generate new fish germplasm or varieties in one generation; thus, the breeding time has been shortened (3, 4). Using this approach, intraspecific hybrids of Atlantic salmon (*Salmo salar*) (5), Chinook salmon (*Oncorhynchus tshawytscha*) (6), and interspecific hybrids of common carp (*Cyprinus carpio*) × red crucian carp (*Carassius auratus*, red var.) (7), Japanese crucian carp (*Carassius cuvieri*) × red crucian carp (8), and channel catfish (*Ictalurus punctatus*) × blue catfish (*I. furcatus*) (9) have been produced as new germplasm or varieties. These hybrids have the potential to be farmed as advanced parents for producing fry with productive advantages or used directly as farming fishes with heterosis (10). Thereinto, the hybrid combination is crucial for the heterosis (11). To date, the researchers could only obtain hybrids with advantages by attempts using different hybrid combinations. Therefore, understanding the genetic mechanism of heterosis is the way to improve the efficiency for hybrid breeding.

Heterosis is the phenomenon in which progeny has improved phenotypes such as higher growth rates, stronger ability to fight diseases, and higher fertility compared to its parents. Since hybrids contain subgenomes from different parents, there is high genetic variation of subgenome and transcriptomic changes (12, 13). In *Arabidopsis* allotetraploids, approximately 5.2%–5.6% genes had expression divergence from the midparent value (MPV) suggesting nonadditive expression (14). Further studies showed that several genes that participated in multiple biological processes including development, organism growth, and hormone regulation were nonadditively expressed (15, 16). In addition, these genes contributed to the heterosis in hybrid plants (17, 18). In hybrid fishes, similar results have been reported, such as Nile tilapia (*Oreochromis niloticus*) × blue tilapia (*O. aureus*) hybrid (15) and blunt snout bream (*Megalobrama amblycephala*) × topmouth culter (*Culter alburnus*) hybrid (19). Generally, it supposes that gene expression in hybrid is equal to MPV showing an additive effect while actually several genes related to growth and anti-disease are nonadditive expression genes. These nonadditive expression genes would affect phenotypes of hybrids leading to heterosis in transcriptome perspective (16). The transcription of gene is regulated by transcription initiation activity, which is controlled by *cis* and *trans* effects (20). In hybrids, the heterozygous genomes provide convenience for parsing the *cis* and *trans* effects. *Cis*-regulatory elements are located in the upstream region of the gene while *trans*-regulatory elements such as transcription factors control transcriptional activities in an intermolecular interaction way (21). Based on these features, the *cis* and *trans* effects could be evaluated by calculating the differential allelic expression value in hybrids and the differential expression between the parents (22). Interpreting the *cis* and *trans* effects of functional genes would provide clues for understanding the genetic mechanism of heterosis from the perspective of nonadditive expression (23).

Growth performance is the key trait for breeding researchers. The growth hormone (GH)/insulin like factor (IGF) axis is the well-known regulatory pathway for controlling growth in fish (24). Besides this axis, other regulators have also been reported recently. Among them, ghrelin secreted in enteroendocrine cells of the gastrointestinal tract has function in growth and appetite regulation (25). Ghrelin also regulates the GH/IGF axis via endocrine regulation (26). A previous study showed that *GH* had higher expression in Nile tilapia × blue tilapia hybrid compared to its parents (27). Additionally, the *GH* showed asymmetric allelic expression in the hybrid tilapia (27). The result showed new clues for the regulation of functional gene expression in hybrid tilapia from transcribing initiation activity level. To date, the connection between the heterozygous genome and expression patterns in hybrid tilapia is far away from systematically understand.

The Nile tilapia (♀) × blue tilapia (♂) hybrid is one of the most popular farmed tilapia varieties in China, due to its fast growth performance and strong resistance to low temperature (28). This tilapia variety also showed higher appetite (29). We suppose that in the hybrid tilapia, the fish may have a special expression pattern of *ghrelin*, which is attributed to the regulation by heterozygous genome. Herein, the *ghrelin* expression in the hybrid tilapia was assayed compared to its parents. The allele-specific expression (ASE) of *ghrelin* in the hybrid tilapia was analyzed. Furthermore, the *cis* and *trans* effects on transcription of *ghrelin* in the hybrid were determined based on the expression results. The present study demonstrated the *ghrelin* expression pattern and its transcriptional regulation, which would aid in understanding the genetic mechanism of advantages in the growth performance of hybrid tilapia.

## 2 Materials and methods

### 2.1 Fishes

The experimental fishes, including Nile tilapia (N), Blue tilapia (B), and their hybrid (NB) were obtained from National Tilapia Breeding Fields of Nanning from Guangxi Academy of Fishery Sciences (Nanning, China). The NB individuals were obtained by interspecific crossing between 20 female N and 10 male B. The experimental fish of N and B were generated by self-mating. All the tested fish were farmed in three adjacent ponds (N, B, and NB were reared in the separate ponds) in Guangxi Academy of Fishery Sciences (Nanning, China). The water and feeding conditions were similar among the ponds. For each kind of tilapia, 200 individuals were reared for 120 days post hatching to adulthood. Before tissue collection, the tested fish were anesthetized by MS-222. The muscle, pituitary, heart, spleen, intestine, brain, ovary, testis, kidney, liver, and stomach were dissected from the fish, frozen in liquid nitrogen, and restored at −80°C. In tilapia, males grow faster than females. Thus, most of the farmed individuals are males that are generated by breeding technology (30, 31). In the present study, considering the differences of growth rate in tilapia between males and females, we only used males for the comparison study for N, B, and NB. The present experiments have been approved by Ethics Committees of Hunan Normal University (Changsha, China).

### 2.2 Ghrelin cloning and phylogenetic analysis

The total RNA from stomach in N and B (3 samples from each species) was isolated using RNAiso Plus (TRIzol) reagent (Takara, Japan) according to the manufacturer’s instruction. The quality and concentration of the extracted RNA were detected by 1% agarose gel and SmartSpec™ Plus Spectrophotometer (Bio-Rad, USA). The cDNA was synthesized by the PrimeScript RT Reagent Kit with gDNA Eraser (Takara, Japan) using 1 μg of total RNA for each sample. The predicted cDNA sequences of *ghrelin* based on N and B genome data were obtained from NCBI. A pair of primers was designed for amplification of *ghrelin* open reading frame (ORF) (**Supplementary Table 1**). The PCR was performed as 94°C for 5 min for initial predenaturation, 30 cycles of 94°C for 30 s, 60°C for 30 s, 72°C for 45 s, and extension at 72°C for 10 min. The PCR products were analyzed by 1.2% agarose gels following extraction and sequenced by Shanghai Sangon Biotechnology Co. Ltd. (Shanghai, China).

The sequenced ORF was compared with *ghrelin* sequences of other species by NCBI blast (https://blast.ncbi.nlm.nih.gov/Blast.cgi). The amino acids were predicted based on the ORF sequences and the alignment of *ghrelin* N and B was constructed using ClustalW (32). The phylogenetic tree was constructed by MRBAYES (Version 3.2.7) with the parameters as prset aamodelpr = VT, mcmcp ngen = 100000000, printfreq = 100, Samplefreq = 100 (33).

### 2.3 Reverse transcriptase PCR

The cDNA from muscle, pituitary, heart, spleen, intestines, brain, ovary, testis, kidney, liver, and stomach in NB was synthesized as mentioned in *Section 2.2*. Reverse transcriptase PCR (RT-PCR) was conducted to detect *ghrelin* expression in these tissues. The primers of *ghrelin* and *β-actin* were designed for the PCR (**Supplementary Table 1**). The parameters of RT-PCR were as follows: 94°C for 5 min for initial predenaturation, 30 cycles of 94°C for 30 s, 60°C for 30 s, 72°C for 45 s, and extension at 72°C for 10 min. The PCR products were detected by 1.2% agarose gels and photographed using a Gel-Doc XR (Bio-Rad, USA).

### 2.4 Quantitative PCR

To compare the *ghrelin* expression in NB and its parents in stomach, the total RNA of stomach from N, B, and NB (*n* = 5 for each kind of tilapia) was isolated and cDNA was synthesized as mentioned in *Section 2.2*. The quantitative PCR (qPCR) was conducted on a PikoReal 96 Real-Time PCR system (Thermo Fisher Scientific, USA). The reagents used for qPCR were TB Green™ Fast qPCR Mix (Takara, Japan) according to the manufacturer’s instruction. The qPCR cycles were as follows: 95°C for 7 min, 40 cycles of 95°C for 5 s, and 60°C for 30 s. After the reaction, the melting curve was used to determine the specificity of PCR amplification. To normalize the results, *β-actin* was introduced as internal reference gene. The *ghrelin* expression levels were calculated using the 2^−ΔΔCT^ method (34). To identify the nonadditive expression, we compared the *ghrelin* expression level in NB and MPV (mean value of the expression level in N and B). If NB ≠ MPV with significant difference, the expression of *ghrelin* was determined as nonadditive expression.

### 2.5 Identification of single-nucleotide polymorphism of the tilapia

By comparing the cDNA sequences of *ghrelin*, two single-nucleotide polymorphism (SNP) sites between the different species were identified. Subsequently, a pair of primers was designed to obtain the sequences with the SNP sites (**Supplementary Table 1**). The PCR was performed and the amplified fragments were sequenced by Shanghai Sangon Biotechnology Co. Ltd. (Shanghai, China). The SNP sites from three individuals of N, B, and NB were compared using the raw data of AB1 file from sequencer (ABI 3730xl, ABI, USA).

### 2.6 Analysis of allele-specific expression in hybrids

Pyrosequencing was used to determine the frequency of allele expression, which was performed by Shanghai Sangon Biotechnology Co. Ltd. (Shanghai, China). The stomach tissues from five different NB individuals were used for the experiment. The primers for targeted sequences containing the identified SNP sites (T/C, C/G) were designed (**Supplementary Table 1**). The PCR conditions were as follows: 95°C for 5 min, 35 cycles of 94°C for 30 s, 60°C for 25–30 s, 72°C for 50 s, and extension of 72°C for 7 min. The PCR production was pyrosequenced by a PyroMark Q96 ID instrument (Qiagen, USA) and the primers are shown in **Supplementary Table 1**.

### 2.7 Evaluation of ghrelin expression in hybrids with different feeding conditions

In order to determine whether different feeding conditions have an effect on *ghrelin* expression in NB, 120 NB individuals (1 month old) were randomly divided into two groups: normal feeding group (control) and fasting group (60 individuals for each group). Before this experiment, the two groups had an adaptation period, during which the hybrids were fed commercial feed with 2% of their body weight. Then, the hybrids in the feeding group were fed with 2% of their weight, while the hybrids in the fasting group were not fed. The stomach of fish from the two groups were collected at 0, 4, 8, 12, 24, and 48 h (*n* = 5). All the individuals were analyzed by qPCR at 0, 4, 8, 12, 24 and 48h to evaluate the mRNA expression of *ghrelin* (*n* = 5). The expression of alleles was assayed by pyrosequencing using the individuals at 0 h and 48 h.

### 2.8 Identification of *cis* and *trans* effects in hybrid tilapia

The determination of *cis* or *trans* effects is based on a previous study (22). The A value is defined as log_2_(NN/BB) (NN = the overall *ghrelin* expression of Nile tilapia, BB = the overall *ghrelin* expression of blue tilapia). Thus, A represents the expression difference between two parents, and also represents the sum of *cis* and *trans* effects. The B value is defined as log_2_ (allelic expression of Nile tilapia in hybrid/allelic expression of blue tilapia in hybrid); hence, B represents the *cis* effect. Thus, A-B represents the trans effect. If (A > 0 and A-B > 0) or (A < 0 and A-B < 0), there were enhancing *cis* and *trans* interactions, and if (A > 0 and A-B < 0) or (A < 0 and A-B > 0), there were compensating *cis* and *trans* interactions.

### 2.9 Statistical analysis

Mean value ± standard deviation was used to present all expression levels. All the statistical analyses were performed by SPSS 16.0 (SPSS Inc., Chicago, USA). The *ghrelin* expression change in stomach among N, B, and NB was compared by one-way ANOVA. The ASE between maternal and paternal sub-genomes was compared using *t*-test, and the ASE between different feeding conditions and times were compared by two-way ANOVA. According to the analysis result, *p* < 0.05 meant there were significant difference between the data.

## 3 Results

### 3.1 Sequences analysis of *ghrelin*

**Figure 1:**
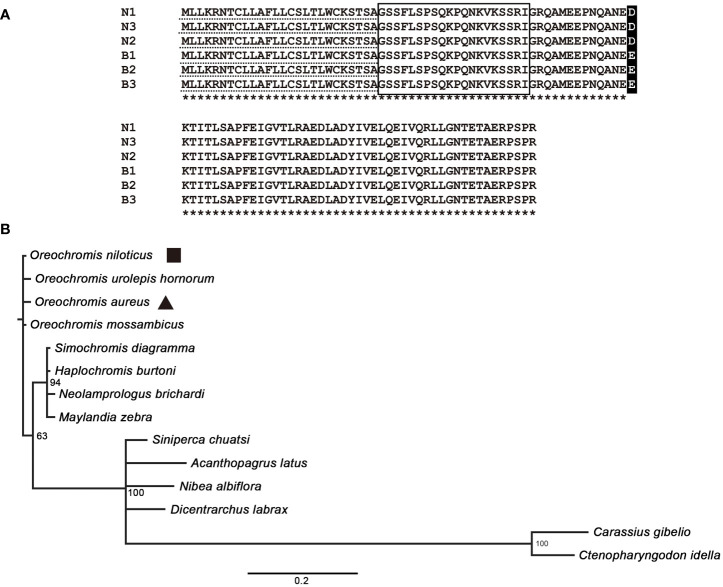
Analysis of preproghrelin protein in tilapia. **(A)** Amino acid sequence alignment of the Nile tilapia and blue tilapia. N1–N3 and B1–B3 represented the three biological replications of Nile tilapia and blue tilapia, respectively. The dotted line showed the signal peptides and the box showed the mature peptide of ghrelin. Asterisks represents the same amino acids. **(B)** Phylogenetic tree analysis using MrBayes 3.2. The sequences were obtained from the Genbank database with Accession Numbers of *Oreochromis niloticus* (XP_003441511.1), *Oreochromis urolepis hornorum* (ABN13418.1), *Oreochromis aureus* (XP_031613045.1), *Oreochromis mossambicus* (BAC55160.1), *Simochromis diagramma* (XP_039885313.1), *Haplochromis burtoni* (XP_005944008.1), *Neolamprologus brichardi* (XP_006807654.1), *Maylandia zebra* (XP_004544940.1), *Siniperca chuatsi* (XP_044033097.1), *Acanthopagrus latus* (XP_036957504.1), *Nibea albiflora* (KAG8007860.1), *Dicentrarchus labrax* (XP_051270674.1), *Carassius gibelio* (XP_052455871.1), and *Ctenopharyngodon idella* (XP_051752346.1).

The ORF sequences of *preproghrelin* from Nile tilapia and blue tilapia were the same in length, which contained 324 nucleotides encoding 107 amino acids. The signal peptides of N and B were both MLLKRNTCLLAFLLCSLTLWCKSTSA. The mature ghrelin peptides from N and B were identical: GSSFLSPSOKPONKVKSSRI. Only one amino acid was different comparing the N and B sequences, which was D/E at the 60th site of the preproghrelin (**Figure 1A**). The phylogenetic tree of preproghrelin suggested that all the Cichlidae fishes were clustered together. The Cyprinidae had the farthest distance to the tilapia. In addition, the species from *Oreochromis* showed higher similarity (**Figure 1B**).

### 3.2 Nonadditive expression of *ghrelin* in hybrid

**Figure 2:**
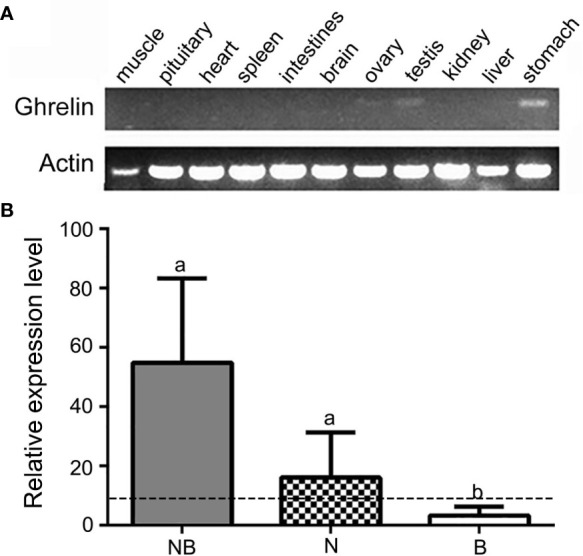
Overall expression of *ghrelin* in hybrid tilapia. **(A)** Tissue distribution of *ghrelin* in hybrid tilapia detected by RT-PCR. **(B)** qPCR analysis of *ghrelin* in the stomach from Nile tilapia (N), blue tilapia **(B)**, and hybrid tilapia (NB). The different lowercase letters on each bar indicated the significant difference (*p* < 0.05) and the dashed line indicated the midparent value (MPV).

The RT-PCR results showed that the *ghrelin* had the highest expression in stomach following testis and ovary while other tissues including muscle, pituitary, heart, spleen, intestine, brain, and liver had no significant signals in NB (**Figure 2A**). Furthermore, we compared the mRNA expression of *ghrelin* in NB and its parents N and B in stomach by qPCR. The results indicated that NB and N had higher expression levels compared to B (*p* < 0.05). Meanwhile, the expression levels of NB were significantly higher than MPV, indicating the nonadditive expression of *ghrelin* in NB (**Figure 2B**).

### 3.3 Identification of the SNP sites between the parents

**Figure 3:**
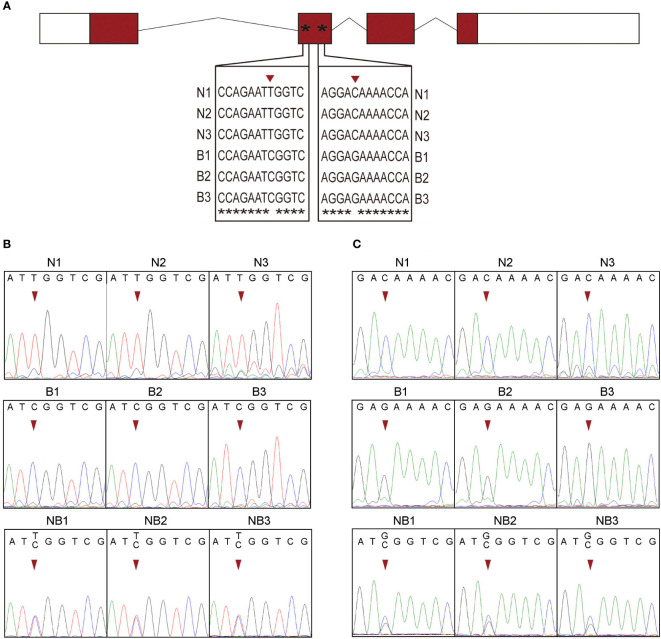
SNP identification form Nile tilapia (N) and blue tilapia (B). **(A)** The two SNP sites were identified from cDNA sequences and displayed using alignment result by ClustalW. Asterisks represents the same nucleotide. **(B, C)** Validation of the two SNP sites by DNA sequencing from N, B, and the hybrid. N1–N3, B1–B3, and NB1–NB3 represented the three biological replications of Nile tilapia, blue tilapia, and hybrid, respectively.

By comparing the cDNA sequences of *ghrelin* in N and B, two SNP sites including T/C and C/G in the second exon of the gene were identified (**Figure 3A**). To determine these two SNP sites, the sequences of genome DNA containing the sites were confirmed from N, B, and NB. By using the raw data of the sequencing, at the T/C site, only T peaks were observed in N and C peaks were found in B. While the NB showed the heterozygosity that contain T and C peaks (**Figure 3B**). At the C/G site, N contained only C peaks and B contained only G peaks, whereas NB had both C and G peaks (**Figure 3C**). These results showed that the T/C and C/G as SNP sites could be used as allelic locus for analyzing allelic-specific expression.

### 3.4 Allele-specific expression of ghrelin in hybrids

**Figure 4:**
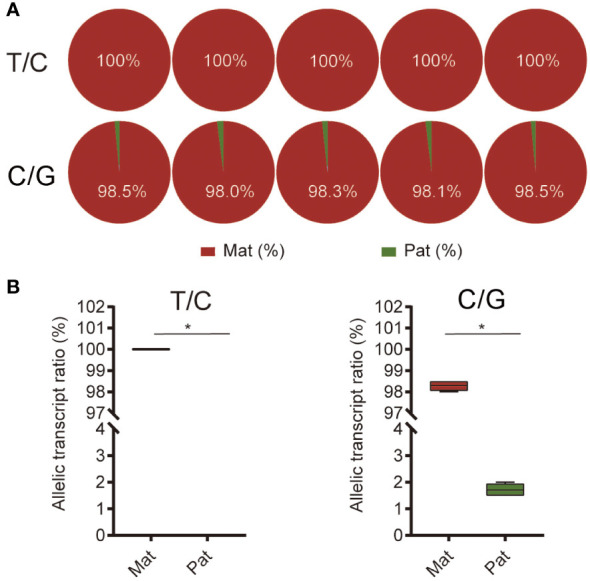
Allele-specific expression (ASE) of *ghrelin* in hybrid. **(A)** The pyrosequencing of *ghrelin* in stomach from hybrid based on the T/C and C/G SNP sites. The five pie plots for each site represented five individuals. **(B)** Allelic transcript ratios of T/C and C/G in hybrid. Mat, maternal; Pat, parental. Asterisks represent values significantly different.

**Figure 5:**
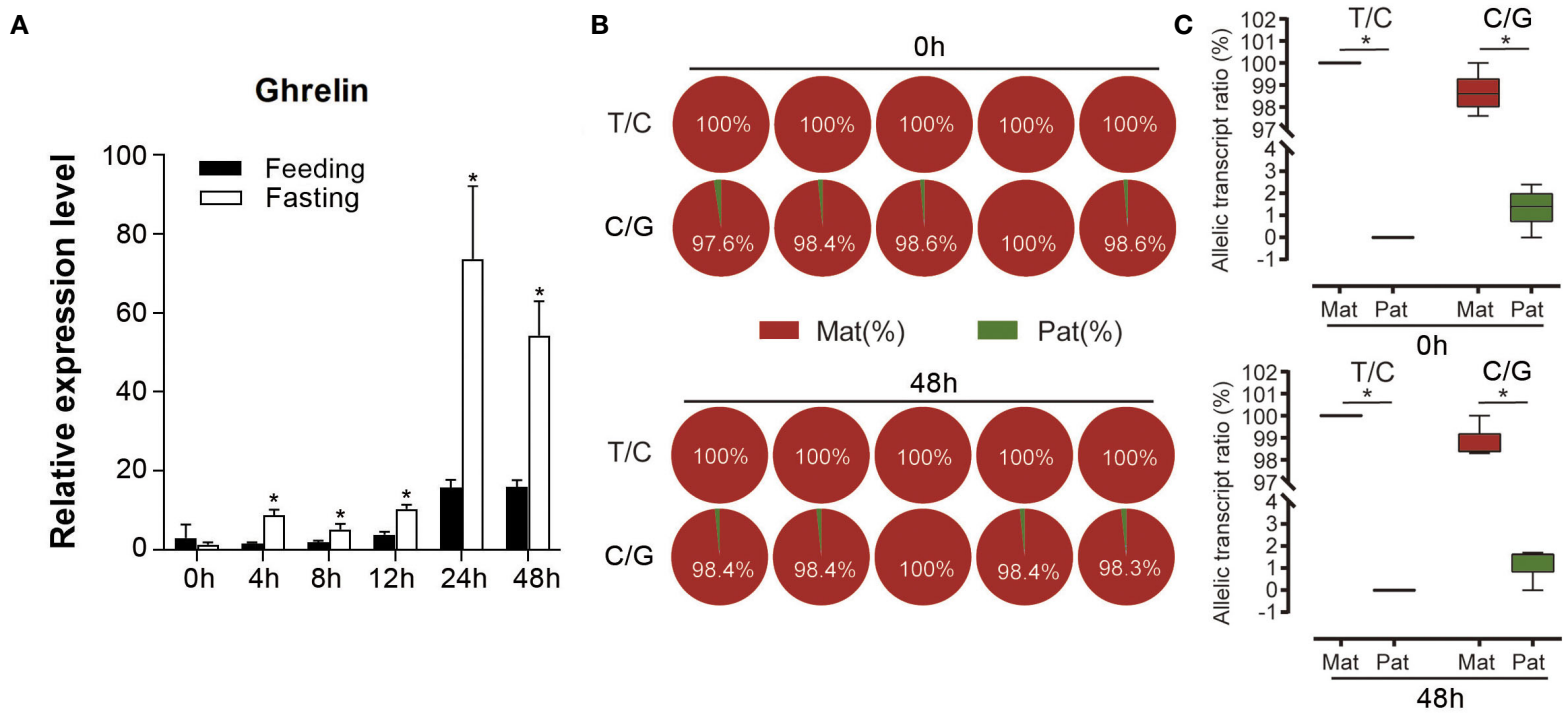
Expression of *ghrelin* in feeding and fasting hybrid. **(A)** Overall expression of *ghrelin* in feeding and fasting hybrid in different time points. Asterisks indicated the significant differences between feeding and fasting group (*p* < 0.05). **(B)** Allele-specific expression (ASE) of *ghrelin* in the fasting group at 0 h and 48 h by pyrosequencing in stomach from hybrid based on the T/C and C/G SNP sites. **(C)** Allelic transcript ratios of T/C and C/G in hybrid. Mat: maternal; Pat: parental.

Pyrosequencing was performed to evaluate the ASE of *ghrelin*. Both the analysis of the two SNP sites suggested the significantly higher expression of maternal alleles than paternal alleles in NB (*p* < 0.05) (**Figure 4**). Furthermore, the feeding and fasting NB were used to determine whether the ASE depends on the feeding condition. For the mRNA expression, *ghrelin* had significantly lower expression in the feeding group compared to the fasting group at 4 h, 8 h, 12 h, 24 h, and 48 h (*p* < 0.05) (**Figure 5A**). The ASE expression of *ghrelin* in the fasting group at 0 h and 48 h was assayed. Both the samples at 0 h and 48 h in the fasting group showed the dominant maternal allele expression with a significant difference (*p* < 0.05) (**Figures 5B, C**).

### 3.5 *Cis*- and *trans*-regulation of *ghrelin* in hybrid

**Figure 6:**
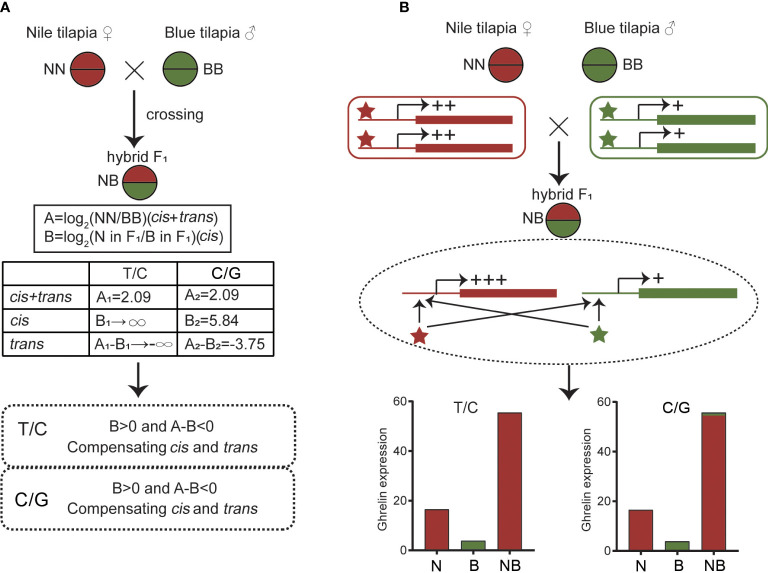
*Cis* and *trans* effects of *ghrelin* in hybrid. **(A)** Determination of *cis* and *trans* effects by calculating the overall expression and allele-specific expression (ASE) values. **(B)** Possible regulatory effects by *cis*- and *trans*-regulators of *ghrelin* in hybrid.

*Cis* and *trans* effects could be determined by calculating the overall expression and ASE of hybrid and its parents. The gene expression divergence defined by the A value could be calculated as log_2_(NN/BB) and the ASE defined by the B value could be calculated as log_2_(N allele in NB/B allele in NB). The result showed that both the T/C and C/G alleles were compensating *cis* and *trans* effects (B > 0 and A-B < 0) in the hybrid, indicating the opposite direction of the regulation (**Figure 6A**).

## 4 Discussion

Ghrelin is the crucial hormone that controls growth and appetite performance (35). Understanding the expression regulation of *ghrelin* may provide valuable information for human health and animals. In fishes, hybridization has been proven as an efficient method for breeding. The hybrids with heterosis could be used for aquaculture, which contributes to the improvement of productivity (36). Hybrid tilapia (NB) is the progeny from intercrossing of N (♀) × B (♂), which has greater growth performance, stronger ability to fight diseases, and anti-low temperature (37, 38). The higher growth rate was reported in previous studies and the non-additive expression of *GH* had been reported before (27). In the present study, we analyzed the *ghrelin* expression and its regulation in NB hybrid. The non-additive expression and ASE suggested that both *cis*- and *trans*- regulation contribute to the appetite and growth advances in NB.

The sequences of preproghrelin shared high similarity in tilapia species including N and B. Only one deduced amino acid was different, suggesting the conserved structure and function. Preproghrelin would be processed to mature ghrelin to perform the function. Intriguingly, the mature ghrelin from N and B were identical. It is known that the ghrelin was secreted by stomach in animals (39). The present results also supported the idea that ghrelin is a gastrointestinal hormone. In addition, our results showed that ghrelin could be produced by ovary and testis. This result suggested the potential function of ghrelin in gonadal development. Ghrelin is a hormone that participates in multiple biological processes including growth controlling and reproductive regulation (40). Previous studies showed that ghrelin interacts with the hypothalamic–pituitary–gonadal axis (HPG axis) to regulate gamete maturation (41, 42). The present study focused on the growth and appetite advances; thus, we further assayed the expression of *ghrelin* in the stomach. In the present study, we found that fasting upregulated *ghrelin* expression in the stomach of NB. Fasting upregulated *ghrelin* expression in fishes has been reported widely, such as in goldfish (*Carassius auratus*), common carp, and grass carp (*Ctenopharyngodon idellus*) (43, 44). The result of the present study also supported the idea that fasting stimulates *ghrelin* secretion in fish gastrointestinal tract, which is similar to the results in other species. As a hormone that rises during fasting, it is reasonable for *ghrelin* to be upregulated by fasting in NB. Furthermore, nonadditive expression of *ghrelin* was found in NB, which is in accordance with the stronger ability to ingest and faster growth in NB. Theoretically, the expression of gene in hybrid is equal to the average of its parents. However, several genes have been found to not abide by this theory, namely, nonadditive expression (17). Nonadditive expression may lead to heterosis (45). This phenomenon could be attributed to the interaction of the two subgenome in hybrid. The subgenome expresses each allele and contributes to the total expression together. In this case, the nonadditive expression of *ghrelin* in NB may be related to allelic gene expression of *ghrelin*.

Two subgenomes were contained in NB as a hybrid. Theoretically, both of the two subgenomes could be expressed genes while the present result showed that ASE of *ghrelin* was found in the stomach from NB. In NB, *ghrelin* from the N subgenome was predominantly expressed while *ghrelin* from the B subgenome could hardly be detected. ASE is common in hybrid, which has been considered as a mechanism for heterosis (46). In addition, ASE provides valuable information for unveiling the mechanism of heterosis for hybrid breeding. The ASE of functional genes has been reported in several plants such as rice (46) and maize hybrids (47). In these studies, the genes that participated in multiple agronomic traits including organism growth, development, and reproduction were ASE, suggesting the underlying effects of ASE on heterosis (46, 47). In contrast, the studies of ASE in hybrid animals were less because it is difficult for interspecific crossing to generate hybrid in higher vertebrates. One of the most well-studied hybrid animals is *Drosophila* (48). Fish interspecific hybrids were also common and ASE in fish has been reported in hybrid *Xiphophorus* (49), cichlid fishes (50), and tilapia (20). In NB hybrid tilapia, GH and IGF-1 predominantly expressed by one subgenome from the parents (27, 37). This is similar to the IGF-2 in human during embryogenesis (51). In human, IGF-2 is the most famous imprint gene, which is related to embryo development, and loss of the expression pattern leads to overgrowth or even death (52). In addition, the feeding conditions did not change the expression trends for NB whether comparing to its parents or the two subgenomes in hybrid. All these clues suggested that the expression pattern of *ghrelin* in NB may lead to the growth advances, which is determined by the genetic rather than the feeding condition.

Since transcriptional activities are regulated by *cis*- and *trans*- regulators (53), the specific expression model of *ghrelin* in NB may due to the changes of the regulators. The present study showed that both *cis* and *trans* effect s were observed in NB. The different *cis*- and *trans*- regulators from parents together affected the expression of *ghrelin* in hybrid. In addition, the compensating *cis* and *trans* effects were found, suggesting the comprehensive regulations. In the hybrids, it has been reported that compensating *cis* and *trans* effects was more common than enhancing *cis* and *trans* effects, only *cis* effects, or only *trans* effects. This may due to the adaptability for heterozygous genome in hybrid. At the beginning of hybridization, two subgenomes from different species were instable. A high probability of recombination, indel, and structural variation exists in the genome from hybrid (54). The variations of genomes lead to changes in *cis* and *trans* factors that contribute to the regulatory changes (55). The *ghrelin* expression pattern in NB is similar, which is affected by the heterozygous genome. Then, *trans*- regulators promoted the transcription of *ghrelin* in the N subgenome while fewer effects were observed in the B subgenome resulting in ASE (**Figure 6B**). Meanwhile, the compressive effects promoted the overall expression of *ghrelin* in NB, indicating the non-additive expression.

## 5 Conclusion

In summary, the present findings suggested the higher expression of *ghrelin* in the hybrid compared to its parents. The maternal bias of *ghrelin* ASE was found in the stomach from NB tilapia, which was not dependent on feeding conditions. The elevated expression and ASE in NB were mediated by compensating *cis* and *trans* effects. The present lines of evidence suggested that the subgenomes in the hybrid contribute to changes of *cis*- and *trans*- regulators, leading to the specific expression pattern in NB.

## Data availability statement

The original contributions presented in the study are included in the article/**Supplementary Material**. Further inquiries can be directed to the corresponding author.

## Ethics statement

All the experiments have been approved by Ethics Committees of Hunan Normal University (Changsha, China). The study was conducted in accordance with the local legislation and institutional requirements.

## Author contributions

HZ: Funding acquisition, Supervision, Writing – original draft. BR: Methodology, Software, Writing – review & editing. CL: Data curation, Software, Writing – review & editing. YZ: Formal Analysis, Funding acquisition, Writing – original draft, Writing – review & editing. YL: Resources, Writing – review & editing. JX: Investigation, Resources, Writing – review & editing.
