# Supplementary material for: Nonadditive and allele-specific expression of ghrelin in hybrid tilapia
Source: Front Endocrinol (Lausanne). 2023 Dec 13;14:1292730. doi: 10.3389/fendo.2023.1292730 (PMC10751329; doi:10.3389/fendo.2023.1292730)
Supplement: Supplementary file 1 [file Table_1.docx]

Table 1 Primers used in the present study

| **Primer name** | **Sequences (5′–3′)** | **Usage** |
| --- | --- | --- |
| Ghrelin-forward | ATCCAGCAGTGGAGACGTTT | Ghrelin cDNA cloning |
| Ghrelin-reverse | TGCTATTTGGCTGATTGATGT |  |
| Ghrelin-RT-forward | GAGCCAAATCAAGCCAATGAG | qPCR for Ghrelin |
| Ghrelin-RT- reverse | TCTGCTCTTAAAGTGACGCCAAT |  |
| β-actin- RT-forward | CCACAGCCGAGAGGGAAAT | qPCR forβ-actin |
| β-actin- RT- reverse | CCATCTCCTGCTCGAAGTC |  |
| pyr-Ghrelin-forward | TCACAGAAACCTCAGAACAAAGT | PCR Amplification of Ghrelin from genome before pyrosequencing |
| pyr-Ghrelin- reverse | GCTCTTAAAGTGACGCCAATT |  |
| pyr-Ghrelin | AAAGTGAAGTCCTCCAGAA | Pyrosequencing |
